# Successful entecavir plus prednisolone treatment for hepatitis B virus-associated membranoproliferative glomerulonephritis: A case report

**DOI:** 10.1097/MD.0000000000014014

**Published:** 2019-01-11

**Authors:** Hiroshi Kataoka, Toshio Mochizuki, Taro Akihisa, Kentaro Kawasoe, Keiko Kawachi, Shiho Makabe, Anri Sawada, Shun Manabe, Masayo Sato, Nobuyuki Amemiya, Michihiro Mitobe, Takafumi Akanuma, Yasuko Ito, Takahiro Inoue, Tomo Suzuki, Katsuomi Matsui, Takahito Moriyama, Shigeru Horita, Mamiko Ohara, Kazuho Honda, Kosaku Nitta

**Affiliations:** a Department of Medicine Kidney Center; b Clinical Research Division for Polycystic Kidney Disease, Department of Medicine, Kidney Center; c Department of Surgical Pathology, Tokyo Women's Medical University, Tokyo; d Department of Nephrology & Hypertension, Kameda Medical Center, Chiba; e Division of Nephrology and Hypertension, Department of Internal Medicine, St. Marianna University School of Medicine, Kanagawa; f Department of Anatomy, Showa University, Tokyo, Japan.

**Keywords:** entecavir, glomerular hypertrophy, hepatitis B surface antigen, hepatitis B virus, membranoproliferative glomerulonephritis, steroids

## Abstract

**Rationale:**

Adult-onset hepatitis B virus-associated membranoproliferative glomerulonephritis (HBV-MPGN) is generally refractory, and an effective treatment for this condition has not been established. The indications for steroids in HBV-MPGN are an important clinical concern.

**Patient concerns:**

A 28-year-old woman with a chronic hepatitis B virus infection developed nephrotic syndrome in her second month of pregnancy, with urinary protein levels of 3 to 10 g/d that continued into her postpartum period. She was a carrier of HBV with HBeAg seroconversion. As her renal impairment could have been a result of pregnancy, we observed her for 10 months postpartum without any intervention. However, spontaneous remission after childbirth was not achieved and urine protein levels were sustained at 1 to 3 g/d. About 10 months after delivery, elevated serum liver enzyme levels were observed.

**Diagnosis:**

Biopsies showed MPGN, with deposition of hepatitis B antigen in the glomeruli, and chronic B-type hepatitis with a severity grade of A1F0. She was diagnosed with HBV-MPGN.

**Interventions:**

The patient was started on entecavir 0.5 mg/d in March 2008. Within 1 month, serum HBV DNA became undetectable; within 3 months, her alanine aminotransferase levels normalized. However, urinary protein excretion did not decrease to <2 g/d. On a second renal biopsy, performed 7 months after entecavir treatment, proliferative lesions of the glomeruli were observed; therefore, prednisolone was started at an initial dose of 30 mg/d.

**Outcomes:**

Her proteinuria improved immediately and prednisolone was tapered over 10 months. A third renal biopsy showed a remarkable resolution of HBV-MPGN, with a significant decrease in mesangial proliferation and immune complex deposition. HBV reactivation was not observed during the prednisolone treatment.

**Lessons:**

Additional prednisolone therapy in combination with antiviral therapy should be considered for refractory HBV-MPGN, with sufficient care taken regarding HBV reactivation.

## 1 Introduction

Despite the remarkable progress in vaccination and antiviral therapy for hepatitis B virus (HBV), approximately one-third of the world's population has serological evidence of a past or current HBV infection.^[1]^ HBV infection during the neonatal period and infancy usually leads to a chronic carrier state. Approximately 240 million people are carriers of chronic HB surface antigen (HBsAg),^[2]^ and approximately 20% to 30% of those in the inactive HBsAg carrier state can undergo spontaneous reactivation of hepatitis B during follow-up.^[3]^ Hepatitis B virus-associated glomerulonephritis (HBV-GN) is one of the extra-hepatic manifestations in patients with chronic HBV infection and asymptomatic carriage of HBV is related to HBV-GN.^[4]^ HBV-GN can manifest in the form of various lesions including membranoproliferative glomerulonephritis (MPGN). However, to date, no effective treatment for adult-onset hepatitis B virus-associated membranoproliferative glomerulonephritis (HBV-MPGN) has been established. Entecavir, which has become available in Japan since 2006, is effective for preventing HBV reactivation, and the emergence of drug resistance for entecavir is rare in nucleoside-naive patients. No case of a 10-year follow-up after the administration of entecavir and prednisolone (PSL) treatment for HBV-MPGN has been reported. Here, we report the case of a patient with concerns of poor renal prognosis due to HBV-MPGN, who was successfully treated with a combination of entecavir and PSL.

## 2 Case presentation

A 28-year-old woman, 2 months postpartum, with a 9-month history of nephritic syndrome was referred to our hospital for evaluation of persistent hematuria and proteinuria in July 2007. She was a carrier of HBV, and virological tests revealed that her serum was HBsAg positive, HBs antibody negative, HBe antigen (HBeAg) negative, and HBe antibody positive (HBeAg seroconversion). She had developed nephrotic syndrome with urine protein 3+, urine occult blood 2+, and hypertension in the second month of pregnancy (October 2006). During late pregnancy, her urinary protein excretion was 3 to 10 g/d, resulting in worsening lower leg edema. After giving birth, her hypertension and bilateral leg edema resolved, but urine protein excretion persisted.

**Figure 1:**
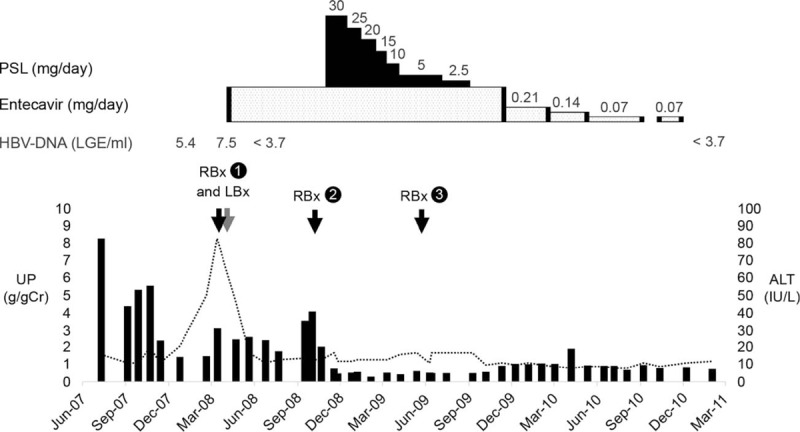
Clinical course. The dotted line indicates serum ALT levels, and the bars indicate the daily urinary protein excretion (g/gCr). Abbreviations: ALT = alanine aminotransferase, HBV = hepatitis B virus, PSL = prednisolone, UP = urinary protein.

At the time she visited our hospital, laboratory findings were as follows: proteinuria (urinary protein: 8.1 g/gCr), microscopic hematuria (urinary occult blood: 3+, 30 to 49 urinary sediment red blood cells per high-power field [HPF]), and hypoproteinemia (serum total protein: 5.3 g/dL; serum albumin: 2.7 g/dL) (Fig. 1, Table 1). She weighed 40.1 kg, with a height of 155.6 cm; her body mass index was 16.6 kg/m^2^. No history of rash, dysuria, jaundice, photosensitivity, joint pains, or previous blood transfusions was reported. On physical examination, her temperature was 36.5 °C; pulse rate, 68/min; respiratory rate, 16/min; and blood pressure, 125/85 mmHg. Other examinations were unremarkable. Her renal and abdominal ultrasound examinations were normal. Laboratory analyses on the first visit showed the following: hemoglobin, 11.0 g/dL; white blood cells, 6300/mm^3^; platelets, 345,000/mm^3^; prothrombin time (INR), 0.79; blood urea nitrogen, 17 mg/dL; serum creatinine, 0.6 mg/dL; sodium, 140 mEq/L; potassium, 3.9 mEq/L; total bilirubin, 0.3 mg/dL; alkaline phosphatase, 135 IU/L; γ-glutamyl transferase, 10 IU/L; aspartate aminotransferase, 26 IU/L; alanine aminotransferase (ALT), 25 IU/L; total protein, 5.3 g/dL; albumin, 2.7 g/dL; total cholesterol, 456 mg/dL; and C-reactive protein, 0.06 mg/dL. Urinalysis revealed that urinary protein excretion was 8.1 g/gCr and urinary red blood cell count was 30 to 49 per HPF. Immunological studies showed that immunoglobulin (Ig) G was 720 mg/dL (normal, 607–1879); IgA, 243 mg/dL (normal, 62–370); IgM, 322 mg/dL (normal, 43–300); C3c, 117.5 mg/dL (normal, 45–102); C4, 22.3 mg/dL (normal, 10–40); and CH, 50 46.2 IU/mL (normal, 30–45). Rheumatoid factor (30.8 IU/mL) and antinuclear antibodies (×80) were both present. Antineutrophil cytoplasmic antibodies and cryoglobulin were not detectable. Based on the hepatitis serologic testing, the patient tested positive for HBsAg, HBeAb, and HBcAb and negative for HBsAb and HBeAg. The serum titer of HBV DNA increased to 7.5 LGE/mL. Tests for hepatitis A virus, hepatitis C virus, and human immunodeficiency virus antibodies showed negative results. The serum level of HBsAg was 16,485 IU/mL and the HBV genotype was type C. She was breastfeeding and her renal impairment could have been secondary to pregnancy; therefore, we observed her after childbirth without providing any treatment. However, spontaneous remission after childbirth was not achieved, and urine protein levels persisted at 1 to 3 g/d. Approximately 10 months after childbirth, elevations in the serum levels of liver enzyme (ALT, 83 IU/L), HBV DNA (7.5 LGE/mL), and urinary protein excretion (2.9 g/gCr) were observed; therefore, a renal biopsy and liver biopsy were performed to decide the treatment strategy.

**Table 1:**
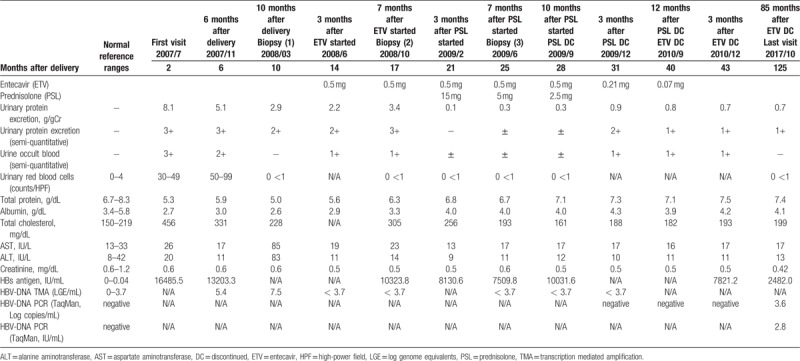
Laboratory findings over the course of the treatment.

**Figure 2:**
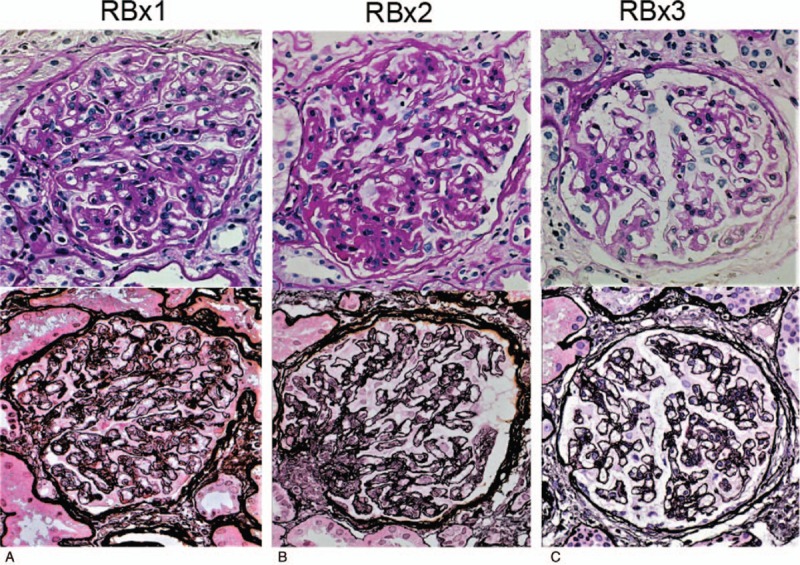
Light microscopy: (upper side, PAS stain; lower side, PAM stain) RBx1: first renal biopsy specimens (A). The glomeruli show large renal corpuscles and MPGN accompanied with PAS-positive deposits, thickening of the glomerular capillary walls, mild increase in mesangial matrix, and mild-to-moderate mesangial hypercellularity. A majority of the glomerular capillary loops demonstrates double contours. PAS stain, ×400; PAM stain, ×400. RBx2: Second renal biopsy specimens (B). The glomeruli show large renal corpuscles and MPGN accompanied with PAS-positive deposits, thickening of the glomerular capillary walls, moderate increase in mesangial matrix, and mild mesangial hypercellularity. A majority of the glomerular capillary loops demonstrates double contours. PAS stain, ×400; PAM stain, ×400. RBX3: Third renal biopsy specimens (C). The glomeruli show MPGN without obvious PAS-positive deposits, thickening of the glomerular capillary walls, increase in mesangial matrix, and mesangial hypercellularity. The glomerular capillary loops demonstrate focal double contours. PAS stain, ×400; PAM stain, ×400. MPGN = membranoproliferative glomerulonephritis, PAM = periodic acid–methenamine–silver, PAS = periodic acid–Schiff, RBx = renal biopsy.

**Figure 3:**
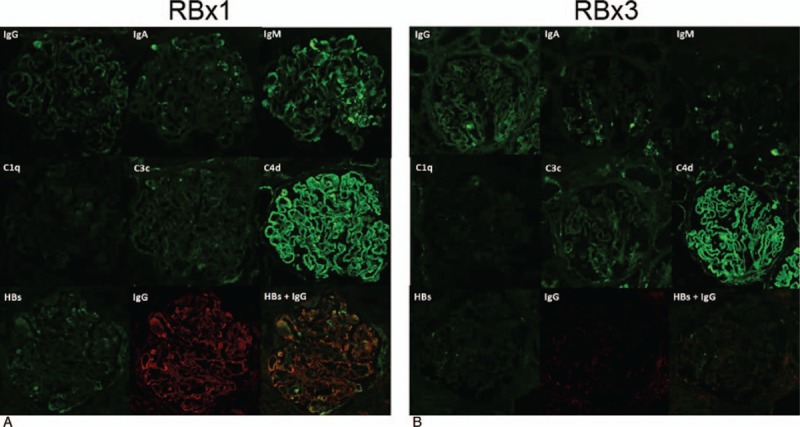
Immunofluorescence microscopy: RBx1: First renal biopsy specimens (A). IgM is positive along the capillary loops in a granular pattern (top right, represented by 2+). IgG and IgA are also present but to a lesser intensity (top left, represented by ±–1+; top middle, represented by 1+). Moderate glomerular C4d staining is observed along the glomerular capillary loops, represented by +2 (middle right). C3c is weakly present (dead center, represented by ±–1+). No C1q is observed in the glomeruli (middle left). Double staining with anti-HBs reagent and IgG showed that the vascular deposits of HBsAg were an integral part of the immunoglobulin and complement deposits (bottom right). These orange-yellow mixtures were resolved into the green fluorescence of HBsAg and the red fluorescence of IgG (bottom left, bottom middle). ×400. RBX3: Third renal biopsy specimens (B). No IgM and IgA depositions are observed in the glomeruli (top right and top middle). Weak and granular pattern of IgG deposition is present (top left, represented by ±). Moderate glomerular C4d staining is observed along the glomerular capillary loops, represented by +2 (middle right). Weak and granular pattern of C3c deposition is present (dead center, represented by ±–1+). No C1q is observed in the glomeruli (middle left). HBsAg was not detected (bottom left). ×400. RBx = renal biopsy.

**Figure 4:**
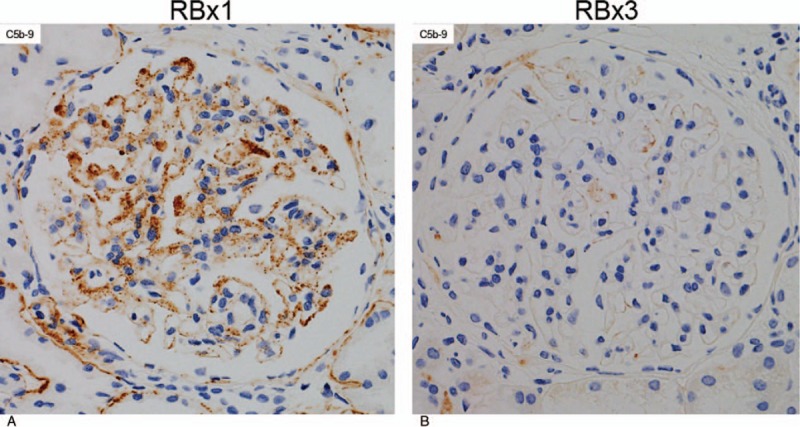
Immunohistochemical staining with Papanicolaou staining: RBx1: First renal biopsy specimens (A). Mild-to-moderate glomerular C5b-9 staining is observed along the glomerular capillary loops (anti-C5b-9). ×400. RBX3: Third renal biopsy specimens (B). No C5b-9 staining is observed (anti-C5b-9). ×400.

**Figure 5:**
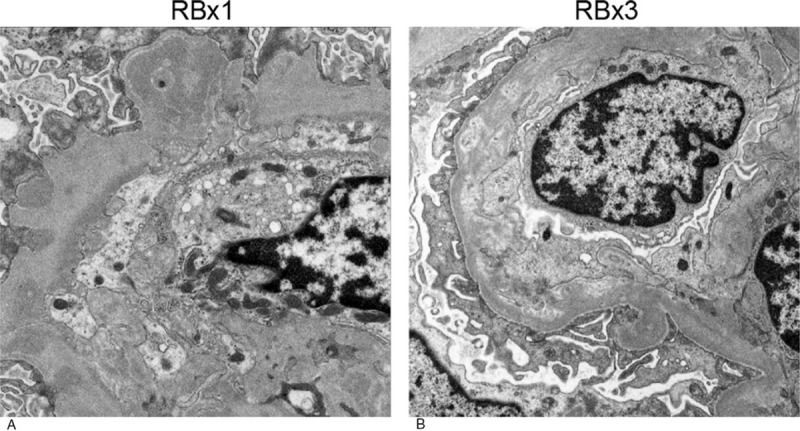
Electron microscopy. RBx1: First renal biopsy specimens (A). The glomeruli show mild foot process fusion of the epithelial cells (represented by 1+). Mesangial interposition is frequently noticed with a marked degree of mesangial proliferation (represented by 3+). Subepithelial (represented by 1+), intramembranous (represented by 2+), and subendothelial (represented by 3+) electron-dense deposits are frequently confirmed, compatible with active MPGN type III. ×2500. RBX3: Third renal biopsy specimens (B). The glomeruli show moderate foot process fusion of the epithelial cells (represented by 2+). Old mesangial interposition is noticed with no mesangial proliferation. Subepithelial (represented by 1+), intramembranous (represented by 2+), and subendothelial (represented by 1+) electron lucent-dense deposits are sparsely confirmed, compatible with inactive MPGN type III. ×2500.

**Figure 6:**
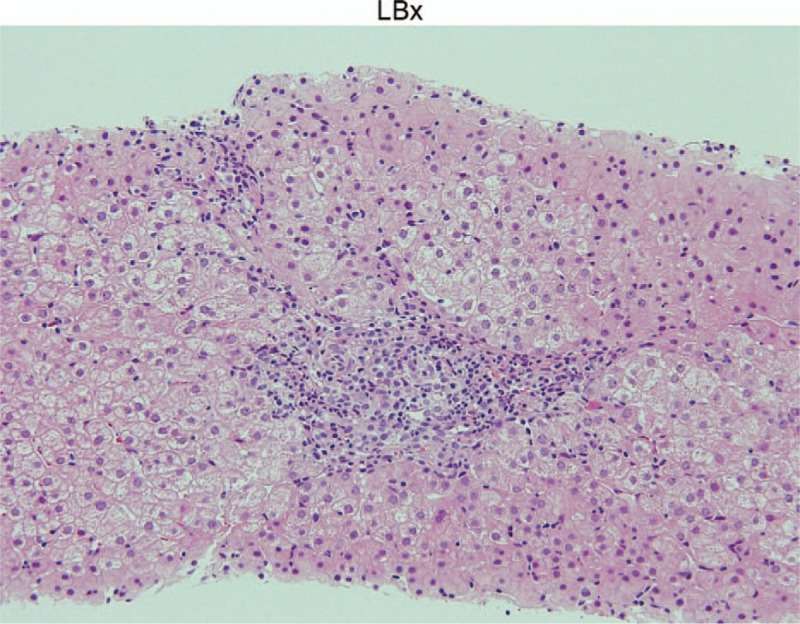
Liver biopsy. The photomicrographs represent liver tissue stained with hematoxylin-eosin stains. There was slight inflammation and lymphocyte invasion in the portal region. The diagnosis was chronic B-type hepatitis and the grade of severity was A1F0. ×100.

The first renal biopsy that was performed in March 2008 revealed large renal corpuscles, glomerular hypertrophy and MPGN (Fig. 2A), which was accompanied by focal deposits of HBsAg in the glomerular capillary wall (Fig. 3, bottom left A). The patient was diagnosed with hepatitis B-related MPGN (HBV-MPGN). The glomeruli had diffuse hypercellularity with a moderate increase in mesangial cells. The glomerular capillary loops were diffusely thickened and double contours were seen on silver staining. On immunofluorescence examination, C1q was not detected, and IgM, IgA, and C4d were detected along the capillary loops in a granular pattern. IgG and C3c were also present but to a lesser extent (Fig. 3). No C1q was observed in the glomeruli. On immunostaining, the deposition of HBsAg and the corresponding immune complexes in the glomeruli were observed (Fig. 3, bottom side A); HBcAg was not detected. Immunohistochemical staining with Papanicolaou staining showed mild-to-moderate glomerular C5b-9 expression along the glomerular capillary loops (Fig. 4A). On electron microscopy, the glomeruli demonstrated mild foot process fusion of the epithelial cells. The capillary loops were thickened with small and large electron-dense deposits on the subepithelial (1+), intramembranous (2+), and subendothelial sides (3+) of the basement membrane, which was compatible with MPGN type III (Fig. 5A). Mesangial interposition was frequently observed with a marked degree of mesangial proliferation (3+). A subsequent liver biopsy demonstrated portal inflammation and changes consistent with mild chronic hepatitis (A1, F0) (Fig. 6).

After liver biopsy, she was started on entecavir at a dose of 0.5 mg once daily in March 2008. Serum HBV DNA became undetectable after 1 month of entecavir treatment; subsequently, the ALT levels normalized within 3 months of entecavir treatment. However, regarding the kidney injury, urine protein excretion did not decrease to <2 g/d. In October 2008 (7 months after entecavir was started), her urinary protein increased to 3 g/d despite showing negative results for HBV DNA. Since no studies at the time had reported on entecavir treatment for HBV-MPGN, we decided to confirm the therapeutic effects with a repeat renal biopsy.

The second renal biopsy was performed 7 months after entecavir treatment; at the time, her laboratory data were as follows: serum ALT (14 IU/L), HBV DNA (<3.7 LGE/mL), and urinary protein excretion (3.4 g/gCr). Light microscopy showed large renal corpuscles, glomerular hypertrophy, and MPGN accompanied by periodic acid–Schiff (PAS)-positive deposits (Fig. 2B). Thickening and double contour of the glomerular capillary walls persisted with a moderate increase in mesangial matrix and mild mesangial hypercellularity. As the active lesions of the glomeruli persisted in the second renal biopsy, prednisolone (PSL) was started at an initial dose of 30 mg/d. After starting PSL treatment, proteinuria improved to 0.1 g/gCr within 3 months and serum albumin remained normal thereafter (Table 1, Fig. 1).

The third renal biopsy was performed 7 months after PSL treatment (5 mg/d of PSL) to evaluate the efficacy; at the time, her laboratory data included serum ALT of 11 IU/L; HBV DNA, <3.7 LGE/mL; and urinary protein excretion, 0.3 g/gCr. The third renal biopsy showed remarkable decrease in the activity of HBV-MPGN with a significant decrease in mesangial proliferation and immune complex deposition compared with previous biopsies (Figs. 2–5). On light microscopy, no obvious PAS-positive deposits and no mesangial hypercellularity were observed (Fig. 2C). Immunofluorescence microscopy showed resolution of the deposition of IgM and IgA in the glomeruli (Fig. 3, top right, and top middle). Immunohistochemical staining with Papanicolaou staining revealed resolution of C5b-9 deposition in the glomeruli (Fig. 4B). On electron microscopy, deposition of electron-dense deposits decreased in the capillary loops, especially in the subendothelial region. It was compatible with inactive MPGN type III, which showed electron lucent-dense deposits (Fig. 5B). Reactivation of HBV was not observed during the PSL treatment, and PSL was discontinued after 10 months.

Although her daily urine protein excretion increased to 0.7 to 0.9 g/d at follow-up, once the PSL treatment was discontinued, protein excretion did not increase to >1 g/d. Entecavir was discontinued after 30 months of therapy. Currently, she is regularly followed-up in the clinic without any treatment and remains free of edema, with normal renal function and serum levels of ALT, albumin, and cholesterol. She also remains HBsAg-positive and HBeAg-negative. The serum levels of HBsAg gradually decreased without antiviral therapy to 2482 IU/mL at the last visit in 2017. Although HBV DNA in her serum was detected again in 2011 (3.3 Log copies/mL, TaqMan), her urine protein was maintained at <1 g/d for >8 years (Table 1, Fig. 1).

## 3 Discussion

The patient in this report was an HBV carrier with a high titer of HBsAg, although she had HBe seroconversion. She developed nephrotic syndrome in association with pregnancy, and urine protein did not decrease until 10 months after childbirth. Treatment with entecavir along with 10 months of glucocorticosteroid therapy contributed to a good 10-year renal prognosis in this patient who had HBV-MPGN with poor prognostic factors; was serologically HBeAb positive, HBcAb positive, and HBsAg positive; and had large renal corpuscles and glomerular hypertrophy in the kidneys.

Generally, HBV-GN is treated with antiviral drugs as the first choice. The patient was receiving entecavir, but its effect was limited. The addition of PSL resulted in a marked decrease in urine protein excretion, and a significant improvement of glomerular status was confirmed on renal biopsy. In clinical settings, determination of the use of steroids in addition to antiviral agents for HBV-GN is often difficult. Furthermore, the criteria for additional steroids have not been established; therefore, the doctors need to contemplate both the characteristics of therapeutic agents and the clinical factors associated with treatment resistance.

For HBV-GN, antiviral drug monotherapy has been generally recommended. Antivirals have proven to be effective in decreasing the urine protein excretion.^[5–7]^ However, studies on antiviral monotherapy for HBV-GN were predominantly comprised of cohorts of patients with hepatitis B virus-associated membranous nephropathy (HBV-MN); therefore, the efficacy of antiviral monotherapy for HBV-MPGN remains unknown. Furthermore, though the quantity of serum HBV-related antigens exacerbate HBV-GN by increasing the deposition of HBV-related antigens, IgA, IgG, IgM, and complement in the glomeruli,^[8]^ the current treatment modalities for HBV infection hardly eliminate HBsAg. Although the current treatment target of hepatitis B infection is a “functional cure,” defined as sustained absence of HBsAg,^[9,10]^ it proceeds at very low rates when treated with entecavir (3% at 96 weeks).^[11]^ As a result, spontaneous HBV reactivation and inflammation occur intermittently throughout the course of chronic HBV infection, leading to exacerbation of HBV-GN. Considering that the natural history of chronic HBV infection is diverse and dependent on complex interactions between the host immune response and HBV, sufficient treatment for HBV-GN was desirable for our patient who was in an “HBeAg-negative immune-active” phase characterized by fluctuating HBV DNA and ALT levels.

In contrast, it has been postulated that use of immunosuppressants leads to unfavorable outcomes in patients with HBV-GN because they suppress the immune system and activate HBV, leading to active replication of HBV and deterioration of kidney lesions.^[12]^ However, in patients who do not respond well to antivirals or in whom antivirals contribute little to proteinuria remission, glucocorticosteroids, along with antivirals, are often empirically used. Considering that the immune complexes and complement have been identified in the glomeruli of HBV-MPGN, it may be reasonable to use glucocorticosteroids to reduce the quantity of immune complex deposition. Zheng et al^[13]^ performed the first meta-analysis of the combined therapy for HBV-GN in 2012. By studying the efficacy and safety of combined antiviral and immunosuppressant therapy, they showed that the combined therapy reduces proteinuria and increases serum albumin concentration without activation of HBV replication or damaging the liver and renal function in adult patients with HBV-GN. Their study showed that patients with HBV-GN treated with combined antiviral and immunosuppressant therapy achieved an overall estimated rate of proteinuria remission of 83%. Their meta-analysis proved the favorable effects of adding immunosuppressant therapy to HBV-GN including HBV-MPGN. However, their observation time was relatively short and included various morphologic histopathological types of glomerular lesions. Therefore, further lesion-specific data regarding the long-term effects of the combined therapy is necessary. Moreover, there is no conclusive evidence to select the suitable treatment for HBV-MPGN. Though entecavir rapid and potent antiviral action allows immunosuppression to be performed effectively without undue effects on HBV replication or risk of chronicity of infection, studies on entecavir plus glucocorticosteroids for HBV-MPGN are quite limited.

Regarding clinical factors associated with treatment resistance, first, we assessed the pathological subtype in HBV-GN. The diagnosis of HBV-GN is usually established by serologic evidence of persistent HBV infection and the presence of glomerular immune complex deposits containing one or more HBV-related antigens (HBsAg, HBeAg, or HB core antigen). HBV infection is associated with various morphologic histopathological types of glomerular lesions,^[8,14]^ including HBV-MN,^[5,15]^ HBV-MPGN, mesangial proliferative nephritis, and focal glomerulosclerosis. Among these, HBV-MPGN in adults has been reported to have a poor prognosis, with mortality or development of end-stage kidney disease,^[5,8]^ while HBV-MN in children has been reported to show spontaneous remission and a good renal prognosis.^[4,5,16,17]^ Zhang et al^[8]^ reported that HBV-MPGN was at its highest prevalence in patients with elevated serum creatinine levels and argued that marked renal impairment was associated with MPGN. We believe that patients should be treated according to their disease and clinical characteristics such as histological type, magnitude of proteinuria, and age. Differences in disease progression between different types of glomerular lesions imply that the therapy for HBV-GN should be adjusted in accordance with the histopathological type of glomerular lesions.

Second, we assessed the combination pattern of serum HBV infectious markers in HBV-GN. Although the pathogenetic mechanisms of HBV-GN are poorly understood, HBV, its immune complexes, and complement components are believed to play an important role in the pathogenesis of HBV-GN. As one of the major mechanisms in the pathogenesis of HBV-GN, immune complexes damage the glomeruli by activating the complement system and cytokines.^[4]^ Antigen-antibody immune complexes against HBs together with complement components are thought to induce glomerular damage. Quan et al^[18]^ advocated the influence of serum HBsAg titers in HBV-GN, and our patient's serum HBsAg levels were high (16,485 IU/mL). Furthermore, Zhang et al,^[8]^ who examined the relationship between serum HBV infectious markers and the staging of renal function in HBV-GN, reported that patients positive for HBsAg, HBeAb, and HBcAb had the highest prevalence of chronic kidney disease stage V (80%). Our patient had just the same pattern of serum HBV markers.

Third, we considered the effect of large renal corpuscles and glomerular hypertrophy on the prognosis in kidney disease. Previously, we have advocated the significance of the large renal corpuscle profile or glomerular hypertrophy in the assessment of renal prognosis and management of kidney diseases.^[19,20]^ Therefore, we believe that large renal corpuscles in the patient's biopsy specimens implied poor renal prognosis. The large renal corpuscles did not disappear with entecavir monotherapy but were eliminated after the addition of glucocorticoids (Fig. 2).

Cumulatively, considering both the unattainable functional cure (HBsAg loss) by therapeutic agents for HBV and her clinical characteristics associated with treatment resistance described above (pathological subtype, the combination pattern of serum HBV infectious markers, high HBsAg titers, and large renal corpuscle/glomerular hypertrophy), prescribing intensive treatment was considered reasonable.

For further assessment, we reviewed the glomerular complement deposition pattern in this patient. In general, immune complex-mediated GN shows bright C4d staining.^[21]^ It was previously reported that positive C4d staining was predictive of a poorer prognosis in IgA nephropathy.^[22]^ C4d is produced not only through the classical pathway but also through the lectin pathway and induces insertion of sublytic levels of the membrane attack complex (C5b-9).^[23]^ Recently, Sethi et al^[21]^ reported that C4d deposition in the glomeruli serves as a positive marker for immune complex-mediated GN. They advocated that the presence of both C1q and C4d is typical of classical pathway activation and that the presence of C4d in the absence of C1q indicated lectin pathway activation in immune complex-mediated GN. Our case was characterized by negative results for C1q deposition and positive results for C4d deposition in the glomeruli in renal biopsies 1 and 3, suggesting the presence of stationary activation of the lectin pathway. Some studies reported that the mannose-binding lectin plays a role in HBV infection. Chong et al^[24]^ showed that the binding of mannose-binding lectin to HBsAg would dependently mediate C4 deposition on HBsAg through the lectin pathway. In our case, C5b-9 deposition in the capillary loops resolved after PSL treatment, but C4d did not (Figs. 3 and 4). C4d is the final degradation product of C4 activation and stays stable on the cell membrane for a long period of time because the covalent bond does not break spontaneously.^[23]^ C4d seems unsuitable for the evaluation of therapeutic reactivity although it has diagnostic utility.

This case report is the first to report the sequential administration of entecavir and glucocorticosteroids for the treatment of HBV-MPGN, with confirmation of the progressive improvements in the histopathology and the urinary protein levels, with follow-up evidence that the disease was well controlled for >10 years. The strengths of this report include the pre-planned treatment and examinations done at intervals of more than half a year to monitor the disease activity and the effects of treatment. Considering that the histological data at the end of the treatment or during the follow-up are generally difficult to obtain, the findings of renal biopsy after treatment are valuable data. These histological end-point data provided in our third renal biopsy reflected the patient's commitment to treatment for HBV-MPGN. The role of entecavir plus PSL in the treatment of HBV-MPGN requires further study. Currently, PSL use in HBV-MPGN is not established, and it should be used with caution in view of the clinical needs according to individual cases. Our report provides clinical indications for using additional steroids when antiviral monotherapy does not prove to be effective alone.

The present report confirms the association between HBV infection and development of nephropathy and supports the efficacy and safety of entecavir plus PSL in patients with HBV-MPGN. Our findings suggest that entecavir monotherapy cannot induce remission of HBV-MPGN in clinical conditions like those in our patient (serologically HBeAb positive, HBcAb positive, and HBsAg positive with high titers, accompanied with large renal corpuscles and glomerular hypertrophy). We suggest that additional PSL therapy can be considered in HBV-MPGN, with sufficient care taken regarding the reactivation of HBV.

## Acknowledgments

The authors express our appreciation to Dr. Takahiro Mochizuki (†Deceased June 25, 2017) for his advice on this work and for his contribution to medical care and medical research in Japan.

## Author contributions

**Conceptualization:** Hiroshi Kataoka, Taro Akihisa, Kentaro Kawasoe, Keiko Kawachi, Shiho Makabe, Anri Sawada, Shun Manabe, Masayo Sato, Nobuyuki Amemiya, Michihiro Mitobe, Takafumi Akanuma, Yasuko Ito, Takahiro Inoue, Tomo Suzuki, Katsuomi Matsui, Takahito Moriyama, Shigeru Horita, Mamiko Ohara, Kazuho Honda.

**Funding acquisition:** Kosaku Nitta.

**Investigation:** Taro Akihisa, Kentaro Kawasoe, Keiko Kawachi, Shiho Makabe, Anri Sawada, Shun Manabe, Masayo Sato, Nobuyuki Amemiya, Michihiro Mitobe, Takafumi Akanuma, Yasuko Ito, Takahiro Inoue, Tomo Suzuki, Katsuomi Matsui, Takahito Moriyama, Shigeru Horita, Mamiko Ohara, Kazuho Honda.

**Supervision:** Taro Akihisa, Kentaro Kawasoe, Keiko Kawachi, Shiho Makabe, Anri Sawada, Shun Manabe, Masayo Sato, Nobuyuki Amemiya, Michihiro Mitobe, Takafumi Akanuma, Yasuko Ito, Takahiro Inoue, Tomo Suzuki, Katsuomi Matsui, Takahito Moriyama, Shigeru Horita, Mamiko Ohara, Kazuho Honda.

**Writing – original draft:** Hiroshi Kataoka.

**Writing – review & editing:** Toshio Mochizuki, Kosaku Nitta.

## References

[1] European Association For The Study Of The Liver. EASL clinical practice guidelines: Management of chronic hepatitis B virus infection. J Hepatol 2012;57:167–85. 22436845 10.1016/j.jhep.2012.02.010

[2] Schweitzer A Horn J Mikolajczyk RT Estimations of worldwide prevalence of chronic hepatitis B virus infection: a systematic review of data published between 1965 and 2013. Lancet 2015;386:1546–55. 26231459 10.1016/S0140-6736(15)61412-X

[3] Tseng TC Huang LR Immunopathogenesis of Hepatitis B Virus. J Infect Dis 2017;216 suppl:S765–70. 29156047 10.1093/infdis/jix356

[4] Johnson RJ Couser WG Hepatitis B infection and renal disease: clinical, immunopathogenetic and therapeutic considerations. Kidney Int 1990;37:663–76. 1968522 10.1038/ki.1990.32

[5] Bhimma R Coovadia HM Hepatitis B virus-associated nephropathy. Am J Nephrol 2004;24:198–211. 14988643 10.1159/000077065

[6] Fabrizi F Dixit V Martin P Meta-analysis: anti-viral therapy of hepatitis B virus-associated glomerulonephritis. Aliment Pharmacol Ther 2006;24:781–8. 16918881 10.1111/j.1365-2036.2006.03041.x

[7] Yang Y Ma YP Chen DP A meta-analysis of antiviral therapy for Hepatitis B virus-associated membranous nephropathy. PLoS One 2016;11:e0160437. 27598699 10.1371/journal.pone.0160437 PMC5012684

[8] Zhang L Meng H Han X The relationship between HBV serum markers and the clinicopathological characteristics of hepatitis B virus-associated glomerulonephritis (HBV-GN) in the northeastern chinese population. Virol J 2012;9:200. 22978266 10.1186/1743-422X-9-200 PMC3499437

[9] Tang LSY Covert E Wilson E Chronic hepatitis B infection: a review. JAMA 2018;319:1802–13. 29715359 10.1001/jama.2018.3795

[10] Lok AS Zoulim F Dusheiko G Hepatitis B cure: from discovery to regulatory approval. Hepatology 2017;66:1296–313. 28762522 10.1002/hep.29323 PMC6294322

[11] Lok AS Trinh H Carosi G Efficacy of entecavir with or without tenofovir disoproxil fumarate for nucleos(t)ide-naive patients with chronic hepatitis B. Gastroenterology 2012;143:619.e1–28.e1. 22643350 10.1053/j.gastro.2012.05.037

[12] Elewa U Sandri AM Kim WR Treatment of hepatitis B virus-associated nephropathy. Nephron Clin Pract 2011;119:c41–9. discussion c49. 21677438 10.1159/000324652

[13] Zheng XY Wei RB Tang L Meta-analysis of combined therapy for adult hepatitis B virus-associated glomerulonephritis. World J Gastroenterol 2012;18:821–32. 22371643 10.3748/wjg.v18.i8.821 PMC3286146

[14] Lee HS Choi Y Yu SH A renal biopsy study of hepatitis B virus-associated nephropathy in Korea. Kidney Int 1988;34:537–43. 3199674 10.1038/ki.1988.215

[15] Gilbert RD Wiggelinkhuizen J The clinical course of hepatitis B virus-associated nephropathy. Pediatr Nephrol 1994;8:11–4. 8142208 10.1007/BF00868249

[16] Wiggelinkhuizen J Sinclair-Smith C Stannard LM Hepatitis B virus associated membranous glomerulonephritis. Arch Dis Child 1983;58:488–96. 6870328 10.1136/adc.58.7.488 PMC1628176

[17] Lai KN Li PK Lui SF Membranous nephropathy related to hepatitis B virus in adults. N Engl J Med 1991;324:1457–63. 2023605 10.1056/NEJM199105233242103

[18] Quan A Portale A Foster S Resolution of hepatitis B virus-related membranoproliferative glomerulonephritis after orthotopic liver transplantation. Pediatr Nephrol 1995;9:599–602. 8580018 10.1007/BF00860947

[19] Kataoka H Ohara M Honda K Maximal glomerular diameter as a 10-year prognostic indicator for IgA nephropathy. Nephrol Dial Transplant 2011;26:3937–43. 21427079 10.1093/ndt/gfr139

[20] Kataoka H Mochizuki T Nitta K Large renal corpuscle: clinical significance of evaluation of the largest renal corpuscle in kidney biopsy specimens. Contrib Nephrol 2018;195:20–30. 29734147 10.1159/000486931

[21] Sethi S Nasr SH De Vriese AS C4d as a diagnostic tool in proliferative GN. J Am Soc Nephrol 2015;26:2852–9. 25991041 10.1681/ASN.2014040406 PMC4625660

[22] Espinosa M Ortega R Sanchez M Spanish Group for Study of Glomerular Diseases (GLOSEN). Association of C4d deposition with clinical outcomes in IgA nephropathy. Clin J Am Soc Nephrol 2014;9:897–904. 24578331 10.2215/CJN.09710913 PMC4011455

[23] Murata K Baldwin WM 3rd Mechanisms of complement activation, C4d deposition, and their contribution to the pathogenesis of antibody-mediated rejection. Transplant Rev (Orlando) 2009;23:139–50. 19362461 10.1016/j.trre.2009.02.005 PMC2797368

[24] Chong WP To YF Ip WK Mannose-binding lectin in chronic hepatitis B virus infection. Hepatology 2005;42:1037–45. 16231358 10.1002/hep.20891

